# General Aspects of Identification, Prevention, and Protection From Medical Errors in Radiation Oncology

**DOI:** 10.7759/cureus.102098

**Published:** 2026-01-22

**Authors:** Marija Živković Radojević, Ksenija Bosnjakovic, Neda Milosavljević, Milos Grujic, Milos Todorovic

**Affiliations:** 1 Department of Clinical Oncology, Faculty of Medical Sciences, University of Kragujevac, Kragujevac, SRB; 2 Center for Radiation Oncology, University Clinical Center Kragujevac, Kragujevac, SRB; 3 Department of Forensic Medicine, Faculty of Medical Sciences, University of Kragujevac, Kragujevac, SRB; 4 Department of Forensic Medicine, University Clinical Center Kragujevac, Kragujevac, SRB

**Keywords:** error prevention, medical error detection, patient safety culture, quality risk management, radiation oncology and clinical oncology

## Abstract

Medical errors represent an inherent risk in all areas of medicine, but in radiation oncology, they are of particular concern due to the complexity of treatment pathways and the potential severity of consequences. While legal systems often equate medical error with negligent treatment, clinicians typically view medical error as an unintended deviation occurring despite adherence to accepted standards of care. This conceptual discrepancy contributes to fear-driven practice, underreporting of adverse events, and missed opportunities for system-level learning. In this in-depth review, we synthesize current evidence on the definitions, mechanisms, and classification of medical errors, with a specific focus on radiation oncology. We critically analyze error patterns across pre-treatment, treatment, and post-treatment phases, emphasizing the interplay between human factors, system vulnerabilities, and technological complexity. Beyond technical considerations, this review provides a novel integrative perspective by linking guideline-based practice, incident learning systems, safety culture, communication strategies, and emerging artificial intelligence-assisted quality assurance approaches. Using Serbia as an illustrative example of a developing country, we highlight how legal frameworks centered predominantly on criminal liability, combined with high clinical workload and limited national reporting systems, may inadvertently discourage transparency and learning from errors. We propose a multidimensional safety framework that integrates protocol harmonization, non-punitive reporting, structured error disclosure, clinician support mechanisms, and advanced technological solutions. By shifting the focus from individual blame to system-based prevention and resolution, this review offers transferable insights for improving patient safety, enhancing clinician well-being and strengthening safety culture in radiation oncology across diverse healthcare settings.

## Introduction and background

Legal context

The definition of medical malpractice varies across legal systems; however, at its core, it refers to situations in which a physician acts contrary to accepted professional rules and generally recognized medical standards [1]. From a clinical perspective, it is crucial to distinguish malpractice from medical error. Medical errors may occur at any stage of physician-patient interaction, beginning with communication and diagnosis and extending through treatment planning, medication administration, or surgical and interventional procedures, even when there is no negligent intent. This distinction is particularly relevant in complex specialties such as radiation oncology where adverse outcomes may arise despite adherence to accepted standards of care. Negligent treatment represents a criminal offense and implies inadequate medical care provided through negligence or intent, resulting in serious bodily harm, deterioration of health, worsening of an existing disease, or death. In contrast, medical error is often understood by clinicians as an unintended deviation occurring within the limits of professional tolerance. Failure to differentiate between these concepts contributes to fear-driven behavior, underreporting of errors, and defensive medicine. Medical errors impose a significant burden on healthcare systems worldwide. They are associated with increased morbidity, mortality, and substantial economic costs. Beyond measurable clinical outcomes, errors have profound psychological consequences for patients, their families, healthcare professionals, and institutions. Emotional responses such as anger, guilt, depression, and even suicidal ideation may arise following real or perceived wrongdoing, especially when legal action is anticipated. Medical errors have a significant psychological impact on clinicians [2,3], which is particularly relevant in radiation oncology given the high cognitive load, reliance on complex technology, and the potential for severe consequences even from rare deviations. Clinicians often internalize errors as personal failure or breach of public trust, leading to reduced professional confidence and increased defensive practices [2-5]. These effects highlight the need not only for error prevention but also for structured systems of identification and damage mitigation in radiation oncology.

The risk of medical error varies considerably between medical specialties. Gynecology and obstetrics are among the most frequently litigated fields [6]. Ramella et al. identified emergency medicine, general surgery, orthopedics, neurosurgery, gynecology and obstetrics, and radiology as high-risk specialties, where fear of litigation often results in redundant diagnostic procedures [7]. In contrast, specialties characterized by fewer emergency situations and strong reliance on standardized diagnostic and therapeutic protocols show a lower frequency of malpractice claims. Oncology disciplines - particularly internal medicine and radiation oncology - stand out due to their protocol-driven workflows and multidisciplinary decision-making processes [7]. Ravlo et al. classified radiation oncology as a medium- to low-risk specialty with respect to the frequency of medical errors [8]. Nevertheless, despite its relatively low litigation rate, radiation oncology ranked fifth among medical specialties in the United States regarding total compensation paid to patients over the past decade [9]. This discrepancy underscores a critical characteristic of radiation oncology: while errors occur infrequently, their potential clinical, psychological, and financial consequences are substantial. Errors in radiation oncology are more often linked to system or device failures rather than individual negligence, reflecting the technological complexity of the field.

Radiotherapy is among the most complex treatment modalities in medicine. It requires intensive cooperation among radiation oncologists, medical physicists, dosimetrists, radiation therapists, engineers, and nursing staff, as well as extensive preparatory actions before, during, and after treatment. A standard course of radiotherapy may involve more than 300 steps and interactions between personnel, equipment, and planning systems [10]. Each additional interaction statistically increases the risk of error, emphasizing the need for robust safety barriers. The primary protective mechanism in radiation oncology consists of multiple, independent verification systems implemented at different stages of the treatment process. These include peer review by radiation oncologists, independent checks by medical physicists and radiation therapists, and automated software-based control systems that prevent progression to subsequent steps until the identified discrepancies are corrected. When relevant radiotherapy protocols are followed, the probability of a serious reportable error is estimated to be below 0.2% per treatment course [11]. Incident learning systems have been shown to improve safety culture and reduce recurrent errors [10,11]. In radiation oncology, where errors may propagate across multiple treatment stages, such systems are particularly valuable for identifying latent system vulnerabilities rather than isolated technical failures. Regulatory frameworks further reinforce the importance of error identification. For example, California Senate Bill 1237 mandates reporting of specific radiotherapy-related events, including irradiation of the wrong patient, treatment of the wrong body part, or delivery of a dose deviating by more than 20% from the prescribed dose [12]. Such legislation promotes transparency, accountability, and system-level learning. It is important to emphasize that not every deviation during radiotherapy constitutes a medical error. Situations such as omission of a bolus, missed fraction, or delayed boost dose that can be subsequently corrected without major clinical consequences are generally not classified as malpractice. However, timely identification and documentation of these deviations enhance patient confidence by demonstrating the existence of institutional mechanisms for recognizing and managing adverse events. Moreover, providing financial compensation to affected patients has been shown to reduce malpractice claims against radiation oncologists by approximately 35% over a 30-year period [12], suggesting that transparent resolution strategies benefit both patients and clinicians.

Types of errors

Radiation oncology requires significant time investment per patient and precise synchronization of multiple professionals and technological systems. Errors may arise from actions of radiation oncologists, medical physicists, dosimetrists, engineers, radiation therapists, or nursing staff [11]. In addition, external consultants involved in patient care may inadvertently contribute to errors. Adverse events may result from equipment malfunction, radioactive source loss or jamming during brachytherapy, or failures in software networks and planning systems. When an error is identified despite preventive measures, prompt reporting to institutional leadership and regulatory authorities, as well as transparent communication with the patient and family, is recommended. Effective management requires clearly defined institutional protocols. Following identification, the immediate supervisor should initiate a formal analysis and assemble a professional response team. Communication with the patient should occur in a calm, controlled environment and be led by the attending physician, who must demonstrate competence, emotional stability, and clarity while presenting harm-mitigation strategies and potential options for compensation. All adverse events should be documented using standardized terminology and reported without delay to institutional leadership and relevant authorities [13]. Standardized reporting enables classification and root-cause analysis, which form the foundation of effective error prevention strategies [10]. International incident learning and patient safety initiatives underscore the importance of standardized reporting, cross-institutional learning, and non-punitive safety cultures. These principles are highly applicable to radiation oncology, where complex workflows and multidisciplinary coordination amplify the benefits of shared learning across centers.

Open discussion of errors at professional meetings and within institutions is essential for improving patient safety. Weingart et al. emphasized that prevention begins with recognition and reporting [14]. Nevertheless, underreporting remains common due to fear of sanctions, litigation, negative publicity, and unfavorable public attitudes toward physicians [7,14,15]. Dossett et al. highlighted the need for structured education that prepares clinicians to receive negative feedback and manage crisis situations [15]. Communication and Optimal Resolution (CANDOR) strategies integrate transparent communication, education, and fair compensation, contributing to reduced preventable errors and improved safety culture [15]. Simultaneously, clinicians should be supported by system-based safeguards that reduce anxiety and fear associated with potential errors [16]. Taken together, these studies provide complementary evidence that medical errors in radiation oncology are predominantly system-driven, reinforcing the need for integrated technical, organizational, and communication-based prevention strategies.

While existing reviews in radiation oncology have primarily focused on technical safety measures, quality assurance processes, and incident learning systems, this review extends beyond a purely technical perspective. Its unique contribution lies in integrating clinical, organizational, psychological, and legal dimensions of medical error, with particular emphasis on the distinction between medical error, accident, and negligent treatment. Furthermore, by incorporating the perspective of developing healthcare systems, this review highlights systemic and legal barriers to transparent error reporting and learning that are underrepresented in the current literature. Through this multidimensional approach, this research advances the existing knowledge by framing medical error in radiation oncology as a system-level challenge rather than an individual failure, thereby offering transferable insights for improving safety culture and patient-centered error management. Given that the available data on this topic are very limited in the European literature, the aim of this review is to critically analyze medical errors in radiation oncology by integrating clinical, organizational, and legal perspectives in order to identify key mechanisms, prevention strategies, and novel system-based approaches to improving patient safety and safety culture.

## Review

Literature search and review methodology

This narrative review was based on a structured literature search of PubMed/MEDLINE and relevant international oncology society websites (National Comprehensive Cancer Network (NCCN), European Society for Radiotherapy and Oncology (ESTRO), American Society for Radiation Oncology (ASTRO), European Organisation for Research and Treatment of Cancer (EORTC), and American Association of Physicists in Medicine (AAPM)). The search included English-language publications that appeared between 2000 and 2024 addressing medical errors, malpractice, patient safety, and error prevention in radiation oncology. Keywords included combinations of “medical error”, “malpractice”, “radiation oncology”, “radiotherapy”, “patient safety”, “incident learning”, and “quality assurance”. Approximately 142 records were screened based on title and abstract, of which about 17 publications were prioritized for full-text review and inclusion. Selection was guided by relevance to radiation oncology practice, focus on error mechanisms or prevention strategies, inclusion of legal or organizational perspectives, and contribution to understanding system-level safety issues. A narrative review approach was chosen because the topic encompasses heterogeneous study designs, guideline documents, legal analyses, and organizational frameworks that are not readily amenable to quantitative synthesis. This method allowed integration of clinical, technical, organizational, and legal evidence and facilitated the identification of patterns, contradictions, and knowledge gaps relevant to patient safety in radiation oncology.

No formal quantitative synthesis was undertaken. Specifically, no statistical pooling, meta-analysis, meta-regression, or estimation of effect sizes was performed. Numerical data cited from the literature (e.g., error frequencies, compensation rates) are reported descriptively as presented in individual studies and were not subjected to comparative or inferential statistical analysis. The findings were synthesized qualitatively using a thematic narrative approach.

The most common mistakes in radiation oncology

Multiple factors may contribute to inappropriate procedures in oncology, making it essential to clearly distinguish expected adverse events from true medical errors (Table 1). In oncology, a significant proportion of patient complaints relate to diagnostic processes. Zylstra et al. analyzed malpractice claims in breast cancer patients and found that younger patients were disproportionately affected, often because their symptoms were initially underestimated [6]. Lawsuits were more likely to be successful when patients were alive at the time of litigation, had metastatic disease at diagnosis, and had a relatively recent onset of symptoms.

**Table 1:** Interpretation of problems in radiation oncology ERBT: External beam radiation therapy; BT: brachytherapy.

Potential problem	Expected event	Error
Disease recurrence	Natural flow	Irradiation with an insufficient dose
		Radiotherapy as the only modality of treatment
		Delay in starting radiotherapy
		Dose delivery accuracy error
		Inadequate positioning
		Inadequate delineation of target volumes
Radiation toxicity	Expected side effect	Non-observance of risk organ limits
		Failure to take into account the total therapeutic dose when combining two radiotherapy modalities (EBRT+BT, EBRT+boost)
		Reirradiation
		Application of the old radiotherapy technique
		Concomitant use of radio potentiators
		Application of high radiotherapy dose

Ravlo et al. analyzed compensation claims filed by women with cervical cancer and reported that approximately 90% of claims were related to the pre-treatment period, particularly delays or errors in establishing a definitive diagnosis [8]. In the treatment and post-treatment phases, claims most commonly involved treatment-related toxicity, loss of fertility, and irreversible complications of chemo-radiotherapy, accounting for 70% of cases [8]. These findings highlight the importance of early diagnostic accuracy and comprehensive patient counseling. Taken together, the studies by Zylstra et al. and Ravlo et al. demonstrate that the pre-treatment phase represents the period of highest malpractice vulnerability in both breast and cervical cancer, primarily driven by diagnostic delays and underestimation of symptoms. While Zylstra et al. emphasize patient-related factors such as younger age and advanced disease at diagnosis, Ravlo et al. further show that inadequate counseling and severe treatment-related toxicity, including fertility loss, substantially contribute to litigation risk, underscoring the need for both early diagnostic vigilance and comprehensive patient communication. In addition, the results presented by Ravlo et al. point to the necessity of very well-planned patient education before starting treatment, so that both the patient and doctors of other specialties can make a clear distinction between the nature of the disease and the expected side effects, as presented in Table 1. In this way, it is possible to potentially reduce patient dissatisfaction and reduce the number of unjustified complaints.

Workload and organizational factors further influence error risk. Tariq et al. demonstrated that increased departmental workload was associated with a higher probability of errors, with seasonal peaks correlating with more severe incidents [13]. Marshall et al. analyzed malpractice claims against radiation oncologists and found that nearly 38% were related to treatment errors, including incorrect needle placement during prostate brachytherapy and radiation myelitis due to inadequate spinal cord protection [17]. Errors in target volume delineation and organ-at-risk identification represented a significant proportion of serious adverse events.

Ganguly et al. addressed automation of error detection in radiation oncology and reported that administrative errors, particularly those related to sociodemographic data, were the most frequent, followed by errors in pre-treatment procedures [10]. Royce et al. demonstrated that the highest compensation payments were associated with head and neck and genitourinary tumors, especially in cases involving incorrect dose or treatment of benign conditions with radiotherapy [9]. These data illustrate that while technical errors may be less frequent, their consequences are often severe. The findings by Tariq et al. and Ganguly et al. highlight the role of system-level pressures and workflow complexity in error generation, while Marshall et al. and Royce et al. illustrate that errors involving dose delivery, target delineation, or high-risk anatomical sites carry the greatest potential for patient harm and compensation claims. In general, these studies reveal a consistent pattern in which pre-treatment and administrative errors occur more frequently, whereas treatment delivery errors, although less common, are associated with disproportionately severe clinical and legal consequences. Importantly, this discrepancy highlights a gap between error frequency and error impact that is not sufficiently addressed in existing safety frameworks.

Mechanisms of protection against medical error in radiation oncology

Adherence to institutional, national, and international protocols is the cornerstone of protection against medical errors in radiation oncology (Table 2). The National Comprehensive Cancer Network (NCCN) guidelines provide an overarching framework for diagnosis, treatment, and follow-up of patients with common malignancies [18]. These guidelines form the basis of more specific recommendations issued by professional societies such as European Society for Radiotherapy and Oncology (ESTRO) and the American Society for Radiation Oncology (ASTRO), the American Brachytherapy Society (ABS) [19-21].

**Table 2:** The most common errors and potential mitigation mechanisms NCCN: National Comprehensive Cancer Network; ESTRO: European Society for Radiotherapy and Oncology; ASTRO: American Society for Radiation Oncology.

The most common mistakes	Mitigation mechanisms	Source
Wrong diagnosis and incorrect determination of the stage of the disease	Adherence to national and international protocols (NCCN, ESTRO, ASTRO, etc.), multidisciplinary approach	Grober and Bohnen [1]
		D'Angelo and Kchir (2)
		Robertson and Long [3]
		Hooker et al. [4]
		Khullar et al. [5]
		Ravlo et al. [8]
		Royce et al. [9]
		Ganguly et al. [10]
Incorrect determination of target volumes and risk organs	Adherence to national and international protocols (NCCN, ESTRO, ASTRO), multidisciplinarni approach	Zylstra et al. [6]
		Ganguly et al. [10]
		Evans and Decker [12]
		Marshall et al. [17]
Incorrect dose delivery and targeting the wrong part of the body or patient	Identification of potential risks; Existence of clear procedures for managing adverse events, including communication with the patient and their family; Standardized reporting: Accurate and detailed error reporting; Training of medical staff on the importance of reporting and methods of reporting; Provide financial compensation to patients	Royce et al. [9]
		Adamson et al. [11]
		Evans and Decker [12]
Technical errors during placement of brachytherapy needles	Provide better education of the brachytherapy team Provide the best possible imaging Provide a procedure for patient care in such situations	Zylstra et al. [6]
		Evans and Decker [12]
Errors in documentation, especially in sociodemographic data	Multiple levels of input control AI control systems Standardized reporting: Accurate and detailed error reporting Training of medical staff on the importance of reporting and methods of reporting	Tanriverdi et al. (2015) [16]
		Evans and Decker et al. (2011) [12]

Additional guidance is provided through contouring atlases and dose-constraint recommendations, including a specific recommendations for the delineation of target volumes and organs at risk (Radiation Therapy Oncology Group (RTOG)), recommendations for permissible dose distribution to organs at risk (Quantitative Analyses of Normal Tissue Effects in the Clinic (QUANTEC)), recommendations for dose calculation by medical physicists (Task Group AAPM (TG-AAPM), as well as technical recommendations from the American Association of Physicists in Medicine [22,23]. Standardized toxicity grading using Common Terminology Criteria for Adverse Events (CTCAE) and European Organisation for Research and Treatment of Cancer (EORTC) criteria enables consistent assessment of acute and late adverse effects [24,25]. Taken together, these guideline frameworks illustrate a multilayered, protocol-driven safety architecture in radiation oncology, where overarching clinical recommendations (NCCN) are operationalized through increasingly specific technical standards for target delineation, dose constraints, calculation, and toxicity assessment (ESTRO, ASTRO, ABS, RTOG, QUANTEC, AAPM, CTCAE, and EORTC). While this structured approach substantially reduces technical variability and error risk, it also reveals a key gap in current practice - namely, the limited integration of organizational, communication, and reporting strategies into guideline-based safety models, as summarized in Table 2. This gap in the current recommendations opens a window to the necessary additional research with the aim of improving the safety and security of oncology patients undergoing radiotherapy, which further highlights the complexity of this branch.

Given the complexity of radiation oncology, continuous professional development and focused education in specific subspecialties are essential. Any deviation from established protocols must be clearly documented and justified, as such deviations represent high-risk situations for error occurrence [6]. Incident learning systems, peer review, and multidisciplinary collaboration further strengthen safety culture.

Automation and artificial intelligence offer emerging opportunities for error detection and risk assessment. Graph-based risk analysis and automated monitoring systems may reduce reliance on human vigilance and improve early detection of deviations [26]. However, technology must complement not replace robust organizational processes and communication frameworks.

As part of measures to reduce the risk of errors during pre-treatment procedures (CT simulations, delineation, creating a radiotherapy plan, verification of accuracy), automated graphics can be introduced for procedures during therapy (positioning, dosimetry, quality assurance procedures, detection of inter- and intra-fraction deviations and toxicity control) and AI systems created for error detection, risk detection and error consequences monitoring [26]. Multiple check systems in radiation oncology, which are more common here than in any other branch of medicine, have provided experts in this field with a high level of safety and protection against errors. However, in developing countries characterized by a large number of patients compared to the number of doctors and devices, this risk is significantly higher [10]. Given the complexity of radiation oncology, continuous professional development and focused subspecialty training are essential, particularly when deviations from established protocols are clinically justified but inherently high-risk and must be carefully documented [6]. Incident learning systems, peer review, and multidisciplinary collaboration strengthen safety culture by addressing errors as system-level events rather than individual failures. Automation and artificial intelligence, including graph-based risk analysis and automated monitoring, enhance early error detection across pre-treatment and treatment workflows but must complement robust organizational processes rather than replace them [26]. Notably, in developing healthcare settings with high patient volumes and limited resources, harmonizing international guidelines into coherent national and institutional protocols is critical to mitigate elevated error risk and improve radiotherapy safety [10]. In addition, the current world protocols should be unified into national and institutional ones, which are created after a detailed analysis of set-up errors, errors during positioning and dose delivery.

The current situation in Serbia

Serbia currently has eight radiotherapy centers applying modern radiotherapy techniques in accordance with international standards. The Radiotherapy Section of the Serbian Medical Society facilitates professional networking and continuous education. National protocols for oncological treatment, medical physics practice, and patient positioning and verification have been developed [27-29].

Despite these advances, several challenges persist. Serbia lacks a specific national registry for radiotherapy-related medical errors and patient complaints, limiting systematic analysis and benchmarking. Verified medical errors are primarily addressed through criminal law, without structured institutional mechanisms for patient compensation. Additionally, standardized procedures for managing and disclosing errors are not uniformly implemented across institutions.

These factors may negatively impact the sense of security among radiation oncology professionals and discourage transparent reporting. Given the complexity of radiation oncology, development of national and institutional systems for error mitigation, reporting, and compensation is essential. 

The Serbian experience is presented as a case study of a developing and transitional healthcare system rather than a country-specific exception. While certain challenges are context-specific, such as the absence of a national radiotherapy error registry and a legal framework primarily focused on criminal liability, many issues are broadly generalizable, including high patient-to-resource ratios, limited non-punitive reporting mechanisms, and variable implementation of national protocols. These observations provide transferable insights relevant to other low- and middle-income or transitional healthcare settings.

## Conclusions

Radiation oncology represents one of the most complex and technology-dependent disciplines in modern medicine, where even minor deviations may result in significant clinical consequences. Although the overall incidence of severe medical errors is relatively low, their potential impact on patients, clinicians, and healthcare systems necessitates continuous attention to prevention, identification, and structured response. This review highlights that medical errors in radiation oncology arise from a dynamic interplay between human factors, system-level vulnerabilities, and external influences. Importantly, it emphasizes the need to clearly distinguish medical error, accident, and negligent treatment, as failure to do so contributes to fear-based practice, underreporting, and missed opportunities for system improvement. Unlike many existing reviews that focus primarily on technical aspects, this article integrates clinical, organizational, legal, and psychological dimensions of error management, providing a more comprehensive understanding of patient safety in radiation oncology.

A key novel insight of this review is the recognition that advanced technology and strict protocols alone are insufficient to ensure safety. Without a supportive safety culture, transparent communication strategies, and non-punitive reporting systems, even the most sophisticated radiotherapy infrastructure remains vulnerable. This issue is particularly pronounced in developing countries, where high workload, limited resources, and legal frameworks centered predominantly on criminal liability further discourage open disclosure and learning from adverse events. The findings underscore the importance of combining guideline-based practice, incident learning systems, education in error disclosure, and emerging technological solutions such as artificial intelligence-assisted quality assurance into a unified safety framework.

Establishing national registries, structured compensation mechanisms, and institutional support systems for clinicians should be viewed as integral components of patient safety, rather than legal or administrative burdens. Ultimately, improving safety in radiation oncology requires a paradigm shift from individual blame to system-based prevention and resolution. By fostering transparency, continuous education, and multidisciplinary collaboration, radiation oncology can further reduce preventable harm, enhance patient trust, and support clinician well-being while maintaining the highest standards of oncological care.
